# Borrmann type IV gastric cancer exhibits distinct clinicopathological features and extremely poor prognosis

**DOI:** 10.3389/fonc.2026.1829364

**Published:** 2026-08-26

**Authors:** Qingyu Lin, Zikang Li, Wei Tang, Jianqiu Liu, Chanyan Huang, Ran Wei, Tianhao Zhang, Yan Qian, Yin Li, Jianhui Chen, Shirong Cai, Jinning Ye, Ertao Zhai

**Affiliations:** 1 Gastrointestinal Surgery Center, the First Affiliated Hospital of Sun Yat-sen University, Guangzhou, Guangdong, China; 2 Laboratory of Surgery, the First Affiliated Hospital of Sun Yat-sen University, Guangzhou, Guangdong, China; 3 Anesthesia Surgery Center, the First Affiliated Hospital of Sun Yat-sen University, Guangzhou, Guangdong, China

**Keywords:** advanced gastric cancer, borrmann classification, borrmann type IV gastric cancer, prognosis, R0 resection

## Abstract

**Background:**

Borrmann type IV gastric cancer (Bor-IV) is clinically recognized as an aggressive phenotype; however, its prognostic burden relative to contemporary TNM staging and its radiological correlates remain incompletely characterized.

**Methods:**

We retrospectively analyzed 1236 patients with advanced gastric adenocarcinoma who underwent radical gastrectomy, including 124 Bor-IV and 1112 Non Bor-IV cases. Clinicopathological features, multimodal imaging characteristics, and survival outcomes were compared. Overall survival (OS) and disease-free survival (DFS) were analyzed using Kaplan-Meier methods and Cox proportional hazards models.

**Results:**

Among 1236 patients, 124 (10.03%) were Bor-IV and 1112 (89.97%) were Non Bor-IV. Bor-IV was associated with more advanced T stage, N stage, pathological stage, larger tumor size, and a higher proportion of signet-ring cell components (all p < 0.001). Multivariate Cox regression analysis confirmed Bor-IV as an independent prognostic factor for poor OS after adjusting for TNM and other clinicopathological factors (HR = 3.837, 95% CI: 2.900–5.080, P < 0.001). Patients with Bor-IV had a median OS of 21.3 months and a 5-year OS rate of 20.24%, significantly worse than Non Bor-IV across all pathological stages, T stages, and N stages. Notably, the survival of Bor-IV patients approached that of stage IV disease. In the Bor-IV subgroup, age > 60 years and advanced T stage were independent predictors of poor OS. Among treatment modalities, only R0 resection was significantly associated with improved DFS and OS.

**Conclusions:**

Bor-IV is characterized by distinctive imaging features and an exceptionally poor prognosis that is not fully captured by conventional TNM staging. Bor-IV may represent a clinically aggressive phenotype within advanced gastric cancer, underscoring the need for refined risk stratification and optimized multimodal treatment strategies in future prospective studies.

## Introduction

Gastric cancer (GC) remains one of the most prevalent malignancies of the digestive system, with a high incidence and mortality rate worldwide (1). Despite notable advances in endoscopic screening techniques and diagnostic imaging in recent years, the clinical detection rate of early-stage GC remains unsatisfactory. As a result, the majority of patients are still diagnosed at an advanced stage, leading to a dismal prognosis, with the overall 5-year survival rate remaining below 50% (1–3). For advanced gastric cancer (AGC), the Borrmann classification, which is based on the macroscopic morphological characteristics of tumors, is one of the most widely used systems in clinical practice and plays an important role in staging, treatment planning, and prognostic evaluation (4). According to this classification, AGC is divided into 4 subtypes: type I (polypoid or mass type), characterized by well-demarcated, protruding lesions within the gastric lumen; type II (ulcerative localized type), presenting as clearly circumscribed ulcerative lesions with slightly elevated margins; type III (ulcerative infiltrative type), featuring poorly defined ulcers with infiltration into surrounding gastric tissues; and type IV (diffusely infiltrative type), which is characterized by diffuse infiltration of the gastric wall layers with ill-defined margins and minimal luminal deformity.

Among these subtypes, Borrmann type IV (Bor-IV) gastric cancer is considered one of the most aggressive and therapeutically challenging forms of AGC, owing to its unique morphological features, distinct biological behavior, and extremely poor prognosis. Previous studies have consistently demonstrated that Bor-IV is frequently associated with diffuse infiltration of the gastric wall, rapid tumor progression, and a strong propensity for serosal invasion, peritoneal dissemination, and distant metastasis, all of which contribute to difficulties in early diagnosis and curative treatment (5–8). One of the most striking characteristics of Bor-IV is its unfavorable long-term outcome. Epidemiological data indicate that the 5-year overall survival (OS) rate for Bor-IV is generally only 5-10%, which is substantially lower than that observed in other Borrmann subtypes (9). Even among patients who undergo curative-intent surgery, the reported 5-year OS rate ranges from only 30% to 38%, and approximately 60-80% of patients experience disease recurrence within 1–2 years after surgery, most commonly in the form of peritoneal metastasis (7, 10, 11). Given these outcomes, surgery alone is often insufficient to achieve durable tumor control. Consequently, perioperative treatment strategies in addition to surgery have received increasing attention. A randomized controlled trial (RCT) demonstrated that neoadjuvant chemotherapy with docetaxel, cisplatin, and 5-fluorouracil/leucovorin significantly increased the radical resection rate and reduced the incidence of positive resection margins compared with postoperative chemotherapy alone in patients with Bor-IV (12).

Moreover, a retrospective analysis of 117 patients with Bor-IV suggested that subtotal gastrectomy combined with postoperative chemotherapy could effectively improve survival and suppress postoperative peritoneal recurrence (13). Hyperthermic intraperitoneal chemotherapy (HIPEC) has also emerged as a promising adjunctive treatment for eradicating microscopic peritoneal disease and has been widely applied in ovarian cancer and peritoneal metastases of gastrointestinal origin (14–19). Collectively, these findings indicate that multimodal treatment strategies may offer survival benefits for selected patients with Bor-IV; however, robust evidence from multicenter, large-scale studies is still lacking, and the optimal therapeutic approach for this aggressive subtype remains to be clearly defined.

Therefore, this study retrospectively collected clinical and pathological data from patients with GC who underwent radical surgical treatment, with the aim of systematically summarizing the clinicopathological characteristics of Bor-IV, identifying independent prognostic factors, and evaluating the impact of different treatment strategies on patient outcomes.

## Patients and methods

### Patients selection

This retrospective study included patients diagnosed with primary GC who underwent radical surgical treatment at the Department of Gastrointestinal Surgery, the First Affiliated Hospital of Sun Yat-sen University between December 2013 and May 2023. The inclusion criteria were as follows (1): histologically confirmed gastric adenocarcinoma (2); receipt of curative surgical treatment, including total gastrectomy or partial gastrectomy, with standard D2 lymphadenectomy, following multidisciplinary team (MDT) assessment confirming resectability, and ultimately achieving R0 resection (3); availability of complete preoperative data, including contrast-enhanced abdominal computed tomography (CT), magnetic resonance imaging (MRI), and gastroscopy, allowing accurate assessment of Borrmann classification; and (4) complete follow-up data with a follow-up duration exceeding 30 days. The exclusion criteria were (1): early stage GC, remnant GC (defined as carcinoma arising in the residual stomach more than 5 years after subtotal gastrectomy performed for benign disease), or recurrent GC (defined as reappearance or relapse of GC after a period of complete remission, clinical cure, or effective disease control following initial treatment) (2); receipt of any preoperative neoadjuvant therapy (including chemotherapy, radiotherapy, or chemoradiotherapy) or adjuvant radiotherapy; and (3) incomplete or inaccurate tumor-node-metastasis (TNM) staging information. Collected clinicopathological variables included sex, age at diagnosis, tumor diameter, T stage, N stage, M stage, pathological stage, presence of a signet-ring cell component, R0 resection status, and treatment modalities (including adjuvant chemotherapy regimens such as SOX, CapeOx, and FOLFOX, as well as immunotherapy).

### Evaluation and classification of borrmann type

Borrmann classification was initially assessed intraoperatively by the operating surgeon and recorded in the operative notes. In this retrospective analysis, Borrmann type was further determined through a comprehensive review of contrast-enhanced abdominal CT, MRI, gastroscopy findings, and surgical records. Based on these evaluations, patients were ultimately classified into four Borrmann types. For subsequent analyses, all patients were categorized into two groups: Non Bor-IV group and Bor-IV group.

### Postoperative follow-up

Postoperative follow-up was conducted either through outpatient visits or telephone interviews by dedicated research personnel, with all data prospectively recorded in the institutional GC database. Follow-up time was calculated from the date of GC diagnosis of each patient, and the final follow-up date was June 22, 2024. Patients were dynamically monitored during follow-up via periodic imaging examinations (including CT and endoscopy) and clinical assessments. Disease-free survival (DFS) was defined as the interval from surgery to the first occurrence of tumor recurrence, death from tumor-related causes, or the last follow-up. Overall survival (OS) was defined as the interval from surgery to death from any cause or the last follow-up. Recurrence events were confirmed by imaging and/or pathological findings and were incorporated into DFS analysis accordingly.

### Ethical statement

This study was approved by the Ethics Committee of the First Affiliated Hospital of Sun Yat-sen University (Approval No (2024).296) and conducted in accordance with the biomedical research guidelines set forth in the Helsinki Declaration.

### Statistical analysis

All statistical analyses were performed using RStudio software (version 4.4.2) and IBM SPSS Statistics (version 25). Continuous variables were compared using Student’s t-test or the Wilcoxon rank-sum test, as appropriate, while categorical variables were analyzed using the chi-square test or Fisher’s exact test. Comparisons among multiple groups were conducted using the Kruskal-Wallis test. Survival analyses were performed using the R packages “survival” and “survminer”. Kaplan-Meier survival curves were generated, and differences between groups were evaluated using the log-rank test. Prognostic factors were assessed using univariate and multivariate Cox proportional hazards regression models. All variables included in the multivariate Cox regression models had no missing data. Cases with missing values in these key covariates were excluded during the case screening process; therefore, the analyses were based on complete-case datasets, and no multiple imputation or other missing-data methods were applied. To ensure the reliability of the multivariate Cox models, the proportional hazards assumption was tested using Schoenfeld residuals, and multicollinearity was evaluated by variance inflation factor (VIF). A two-sided p value < 0.05 was considered statistically significant.

## Results

### Baseline clinicopathological characteristics

**Figure 1:**
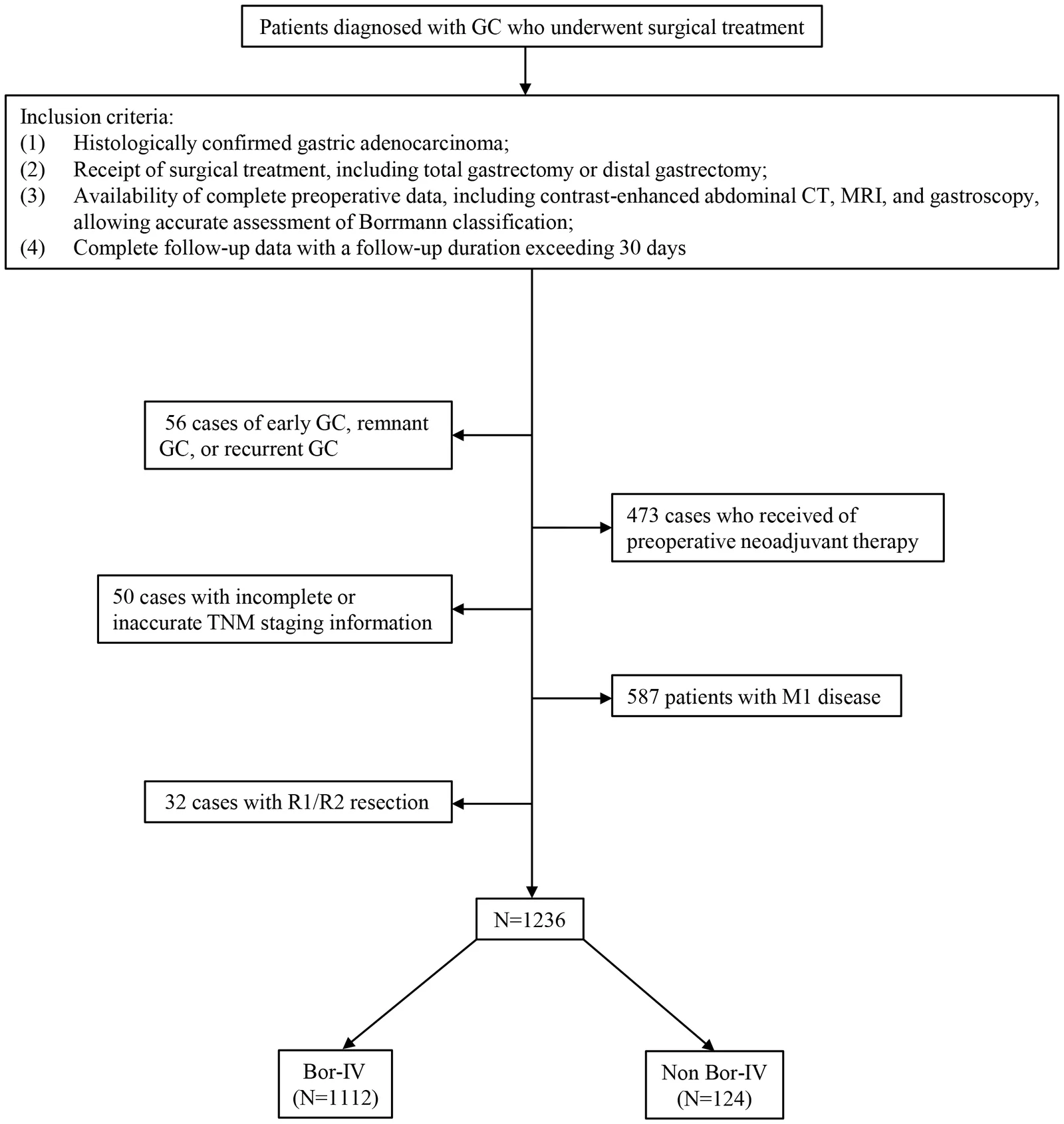
Flowchart of patient inclusion.

A total of 1236 patients who met the predefined inclusion and exclusion criteria were included in the final analytic cohort. According to the Borrmann classification, 1112 patients (89.97%) were assigned to the Non Bor-IV group, which comprised type I (3.60%), type II (21.67%), and type III (74.73%) cases, whereas 124 patients (10.03%) were classified as Bor-IV group (**Figure 1**).

**Figure 2:**
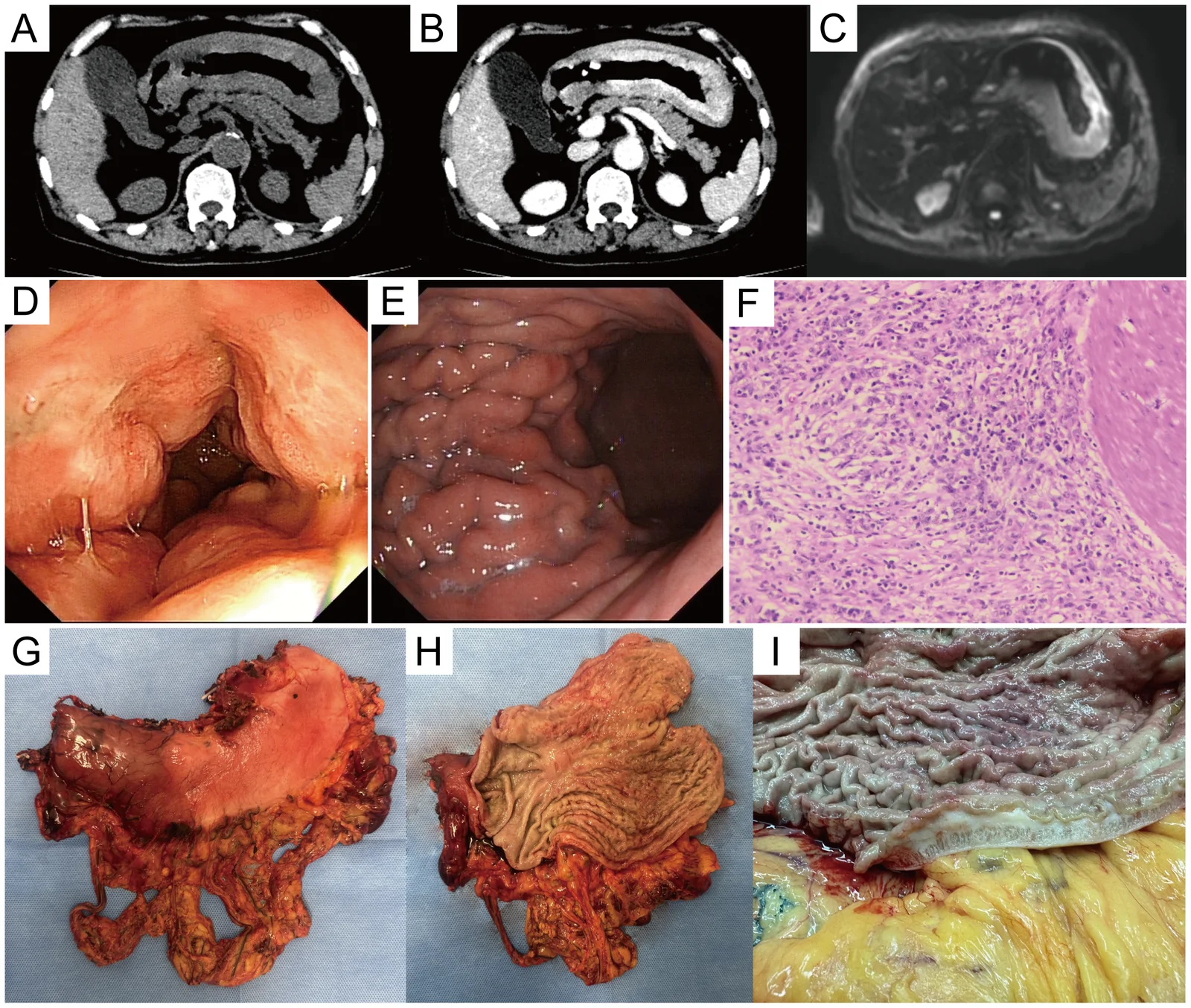
Typical clinicopathological features of Bor-IV. **(A, B)** Abdominal CT demonstrates diffuse full-thickness gastric wall thickening on non-contrast images and a layered enhancement pattern on contrast-enhanced scans. **(C)** Diffusion-weighted MRI shows the characteristic “sandwich sign” of the infiltrative gastric lesion. **(D, E)** Gastroscopy reveals luminal narrowing and marked rigidity of the gastric wall with poor distensibility. **(F)** Hematoxylin and eosin staining shows scattered signet-ring cell components within the tumor tissue. **(G-I)** Gross examination of the resected specimen demonstrates diffuse circumferential wall thickening, a pale and rigid gastric wall, and poorly defined tumor margin.

In Bor-IV group, typical radiological and macroscopic features reflecting diffuse infiltrative growth were consistently observed (**Figure 2**). Contrast-enhanced abdominal CT commonly demonstrated circumferential full-thickness thickening of the gastric wall with a layered enhancement pattern, suggesting extensive submucosal infiltration and desmoplastic reaction. On diffusion-weighted MRI, lesions frequently exhibited the typical “sandwich sign”, characterized by hyperintense signals in the thickened gastric wall, indicating high cellular density and restricted diffusion. Endoscopic examination revealed marked luminal narrowing, poor gastric distensibility, and rigid gastric walls with relatively inconspicuous mucosal ulceration. Gross pathological examination of resected specimens further confirmed these findings, showing diffuse circumferential wall thickening, pale and firm gastric walls, and ill-defined tumor boundarie. These multimodal imaging and macroscopic features collectively highlight the aggressive infiltrative nature and diagnostic challenges associated with Bor-IV.

Significant differences in clinicopathological characteristics were observed between the Non Bor-IV and Bor-IV groups (**Table 1**). The Bor-IV cohort showed a lower proportion of male patients compared with the Non Bor-IV cohort (52.42% vs. 68.71%, p < 0.001), while median age at diagnosis was comparable between the two groups (62.5 vs. 61.0 years, p = 0.994). Tumor size was markedly larger in the Bor-IV group, with tumors >5 cm observed in 64.52% of patients, compared with 24.64% in the Non Bor-IV group (p < 0.001). Tumor location also differed significantly between the two groups: Bor-IV tumors were predominantly located in the middle third of the stomach (60.48% vs. 22.75%) or involved the entire stomach (16.13% vs. 0.81%), whereas Non Bor-IV tumors were more frequently located in the lower third (44.42% vs. 13.71%) or upper third (32.01% vs. 9.68%) (p < 0.001). Regarding tumor invasion depth, advanced T stage was more frequent in Bor-IV patients, with higher proportions of T4a (58.87% vs. 43.17%) and T4b disease (19.35% vs. 10.16%) (p < 0.001). Similarly, lymph node metastasis was more extensive in the Bor-IV cohort, characterized by increased rates of N3a (35.48% vs. 19.15%) and N3b involvement (25.81% vs. 8.90%), and a reduced proportion of node-negative disease (15.32% vs. 30.94%) (p < 0.001). Consistently, advanced pathological stage was more prevalent in Bor-IV patients, with stage III disease accounting for 83.87% compared with 59.17% in the Non Bor-IV group (p < 0.001). In terms of histological features, signet-ring cell components were significantly more common in Bor-IV tumors (21.77% vs. 10.52%, p < 0.001), whereas differentiation grade was similarly distributed between the two groups, with poorly differentiated tumors accounting for approximately 80% in both cohorts (p = 0.776). And the type of gastrectomy differed markedly between the two cohorts, total gastrectomy was performed in 80.65% of Bor-IV patients, compared with only 1.44% in the Non Bor-IV group, whereas partial gastrectomy was the predominant procedure in Non Bor-IV patients (98.56% vs. 19.35%, p < 0.001). In contrast, no significant difference was observed in the proportion of patients receiving chemotherapy between the two groups (61.29% vs. 64.39%, p = 0.560).

**Table 1:** Comparison of clinicopathological characteristics between the Non Bor-IV and Bor-IV groups.

Characteristics	Non Bor-IV cohort		Bor-IV cohort		P value
	(N = 1 112)		(N = 1 24)		
	N	Proportion (%)	N	Proportion (%)	
Sex					**<0.001**
Male	764	68.71	65	52.42	
Female	348	31.29	59	47.58	
Age, M (P25, P75), years	61.0 (52.0, 66.0)		62.5 (54.0, 69.8)		0.994
Tumor size, cm					**<0.001**
Not over 5	838	75.36	44	35.48	
Over 5	274	24.64	80	64.52	
Tumor location					**<0.001**
Upper	356	32.01	12	9.68	
Middle	253	22.75	75	60.48	
Lower	494	44.42	17	13.71	
Total	9	0.81	20	16.13	
Borrmann type					**<0.001**
I	40	3.60	0	0.00	
II	241	21.67	0	0.00	
III	831	74.73	0	0.00	
IV	0	0.00	124	100.00	
T stage					**<0.001**
T2	186	16.73	5	4.03	
T3	333	29.95	22	17.74	
T4a	480	43.17	73	58.87	
T4b	113	10.16	24	19.35	
N stage					**<0.001**
N0	344	30.94	19	15.32	
N1	218	19.60	12	9.68	
N2	238	21.40	17	13.71	
N3a	213	19.15	44	35.48	
N3b	99	8.90	32	25.81	
Stage					**<0.001**
I	102	9.17	2	1.61	
II	352	31.65	18	14.52	
III	658	59.17	104	83.87	
Differentiation grade					0.776
Well-to-moderately	221	19.87	26	20.97	
Poorly	891	80.13	98	79.03	
					
Signet ring cell					**<0.001**
No	995	89.48	97	78.23	
Yes	117	10.52	27	21.77	
Gastrectomy					**<0.001**
Total	16	1.44	100	80.65	
Partial	1096	98.56	24	19.35	
Adjuvant chemotherapy					0.560
No	396	35.61	48	38.71	
Yes	716	64.39	76	61.29	

Bold values indicate statistical significance (P < 0.05).

### Cox regression analysis of independent prognostic factors in the overall cohort

To further evaluate the independent prognostic value of Bor-IV beyond TNM staging, we performed univariate and multivariate Cox proportional hazards regression analyses in the entire cohort, incorporating Borrmann classification along with other potential prognostic factors, including sex, age, tumor size, T stage, N stage, signet ring cell, and adjuvant chemotherapy (**Table 2**).

**Table 2:** Cox regression analysis of independent prognostic factors in the overall cohort.

	Univariate		Multivariate	
	HR (95% CI)	P value	HR (95% CI)	P value
Sex				
Male	1 [ref]			
Female	1.162 (0.926-1.460)	0.195		
Age, years				
Not over 60	1 [ref]		1 [ref]	
Over 60	1.280 (1.026-1.596)	**0.029**	1.050 (0.826-1.337)	0.691
Tumor size, cm				
Not over 5	1 [ref]		1 [ref]	
Over 5	1.871 (1.499-2.335)	**<0.001**	1.156 (0.893-1.498)	0.272
Borrmann type IV				
No	1 [ref]		1 [ref]	
Yes	4.702 (3.640-6.074)	**<0.001**	3.837 (2.900-5.080)	**<0.001**
T stage				
T2+T3	1 [ref]		1 [ref]	
T4	1.934 (1.523-2.457)	**<0.001**	1.466 (1.143-1.879)	**0.003**
N stage				
N (–)	1 [ref]		1 [ref]	
N (+)	3.030 (2.234-4.109)	**<0.001**	2.521 (1.848-3.439)	**<0.001**
Signet ring cell				
No	1 [ref]			
Yes	1.259 (0.913-1.735)	0.160		
Adjuvant chemotherapy				
No	1 [ref]			
Yes	0.959 (0.766-1.200)	0.713		

Bold values indicate statistical significance (P < 0.05).

Univariate analysis identified several factors associated with poorer OS, including older age, larger tumor size, Bor-IV, advanced T stage, and positive lymph node status. Multivariate analysis demonstrated that Bor-IV remained an independent prognostic factor for worse OS after adjusting for age, tumor size, T stage, N stage, and other covariates (HR = 3.837, 95% CI: 2.900-5.080, P < 0.001). In addition, advanced T stage (HR = 1.466, 95% CI: 1.143-1.879, P = 0.003) and positive lymph node status (HR = 2.521, 95% CI: 1.848-3.439, P < 0.001) were also identified as independent predictors of poor prognosis.

### Prognostic comparison between Bor-IV and Non Bor-IV according to pathological stage

**Figure 3:**
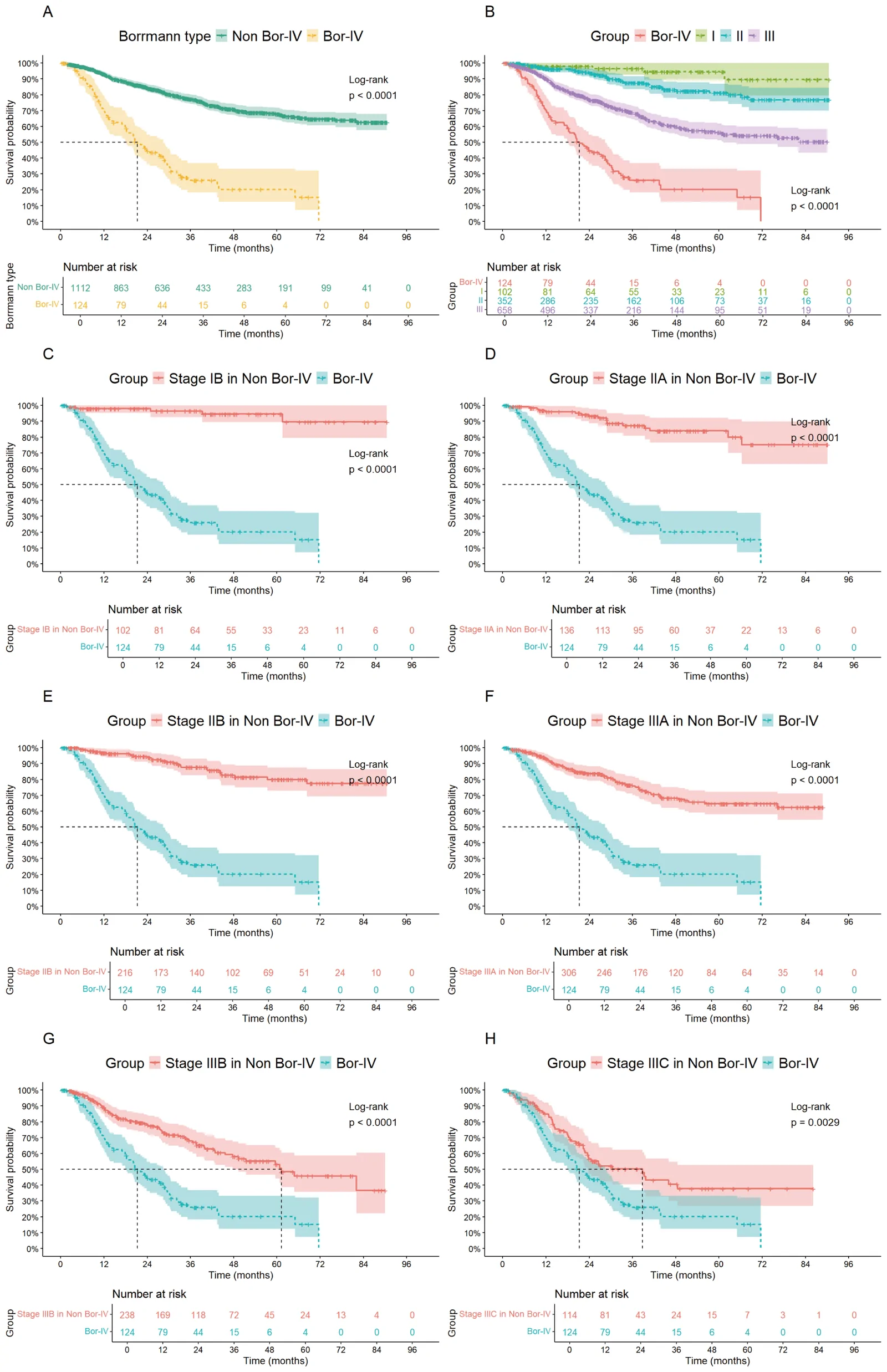
OS comparison between Bor-IV and Non Bor-IV groups with stratification by pathological stage. **(A)** OS comparison between Bor-IV and Non Bor-IV groups. **(B)** OS comparison between Bor-IV group and Non Bor-IV groups at stage I, II and III. **(C–H)** Comparison with stage IB, IIA, IIB, IIIA, IIIB and IIIC in Non Bor-IV groups.

In the overall cohort, patients with Bor-IV exhibited markedly worse survival outcomes than those with Non Bor-IV disease (**Figure 3A**). The 5-year OS rate in the Non Bor-IV group was 67.80%, whereas patients in the Bor-IV group had a median OS of 21.3 months and a 5-year OS rate of 20.24%. Kaplan-Meier analysis demonstrated a significantly poorer prognosis in the Bor-IV group (p < 0.001).

Across different pathological stages, Bor-IV was associated with significantly worse OS than Non Bor-IV group (**Figures 3B–H**). This survival disadvantage remained evident for stage IB disease (5-year OS 94.59%), stage IIA (5-year OS 83.93%), stage IIB (5-year OS 80.01%), stage IIIA (5-year OS 64.74%), IIIB (median OS 61.3 months; 5-year OS 53.04%) and stage IIIC (median OS 38.8 months; 5-year OS 37.70%) (all p < 0.05).

### Cox regression analysis of prognostic factors in the Non Bor-IV and Bor-IV cohort

Univariate Cox regression analysis identified advanced tumor burden as factors associated with poorer survival outcomes mainly in both groups. In Non Bor-IV cohort, larger tumor size, advanced T stage and lymph node metastasis were associated with reduced OS. And in Bor-IV cohort, besides advanced age, adverse pathological features-particularly advanced T stage and nodal involvement-were associated with unfavorable prognosis.

Multivariate Cox regression analysis further delineated distinct prognostic patterns between these two groups. In Non Bor-IV group, advanced T stage (HR = 1.375, 95% CI 1.050-1.801, p = 0.021) and positive lymph node status (HR = 2.760, 95% CI 1.950-3.907, p < 0.001) remained independent predictors of poor survival. Among patients with Bor-IV, multivariate analysis identified being over 60 years old (HR = 1.658, 95% CI 1.041-2.639, p = 0.033) and advanced T stage (HR = 2.318, 95% CI 1.234-4.355, p = 0.009) as the independent worse prognostic factors. Although lymph node positivity was significant in univariate analysis (HR = 2.147, p = 0.032), it did not retain independent significance after adjustment (HR = 1.801, p = 0.103) (**Table 3**). The C-index of the multivariate model was 0.629 (95% CI: 0.596–0.663) for the Non Bor-IV cohort and 0.612 (95% CI: 0.549–0.674) for the Bor-IV cohort, indicating acceptable discriminative ability.

**Table 3:** Cox regression analysis of prognostic factors in the Non Bor-IV and Bor-IV groups.

Variables	Non Bor-IV cohort				Bor-IV cohort			
	Univariate		Multivariate		Univariate		Multivariate	
	HR (95% CI)	p value	HR (95% CI)	p value	HR (95% CI)	p value	HR (95% CI)	p value
Sex								
Male	1 [ref]				1 [ref]			
Female	0.952 (0.723-1.253)	0.724			1.021 (0.657-1.585)	0.927		
Age, years								
Not over 60	1 [ref]				1 [ref]		1 [ref]	
Over 60	1.215 (0.943-1.566)	0.133			1.599 (1.014-2.523)	**0.044**	1.658 (1.041-2.639)	**0.033**
Tumor size, cm								
Not over 5	1 [ref]		1 [ref]		1 [ref]			
Over 5	1.380 (1.051-1.811)	**0.021**	1.175 (0.892-1.549)	0.251	1.452 (0.893-2.360)	0.132		
T stage								
T2+T3	1 [ref]		1 [ref]		1 [ref]		1 [ref]	
T4	1.617 (1.240-2.109)	**<0.001**	1.375 (1.050-1.801)	**0.021**	2.360 (1.271-4.382)	**0.007**	2.318 (1.234-4.355)	**0.009**
N stage								
N (-)	1 [ref]		1 [ref]		1 [ref]		1 [ref]	
N (+)	2.977 (2.112-4.196)	**<0.001**	2.760 (1.950-3.907)	**<0.001**	2.147 (1.069-4.312)	**0.032**	1.801 (0.887-3.654)	0.103
Signet ring cell								
No	1 [ref]				1 [ref]			
Yes	1.013 (0.670-1.532)	0.952			1.166 (0.687-1.977)	0.569		
Adjuvant chemotherapy								
No	1 [ref]				1 [ref]			
Yes	0.923 (0.714-1.195)	0.544			1.005 (0.633-1.595)	0.984		

Bold values indicate statistical significance (P < 0.05).

### Prognosis of Bor-IV versus Non Bor-IV patients stratified by TNM stage

Collectively, subgroup analyses revealed that patients with Bor-IV had markedly inferior survival compared with Non Bor-IV patients across all T and N stages.

**Figure 4:**
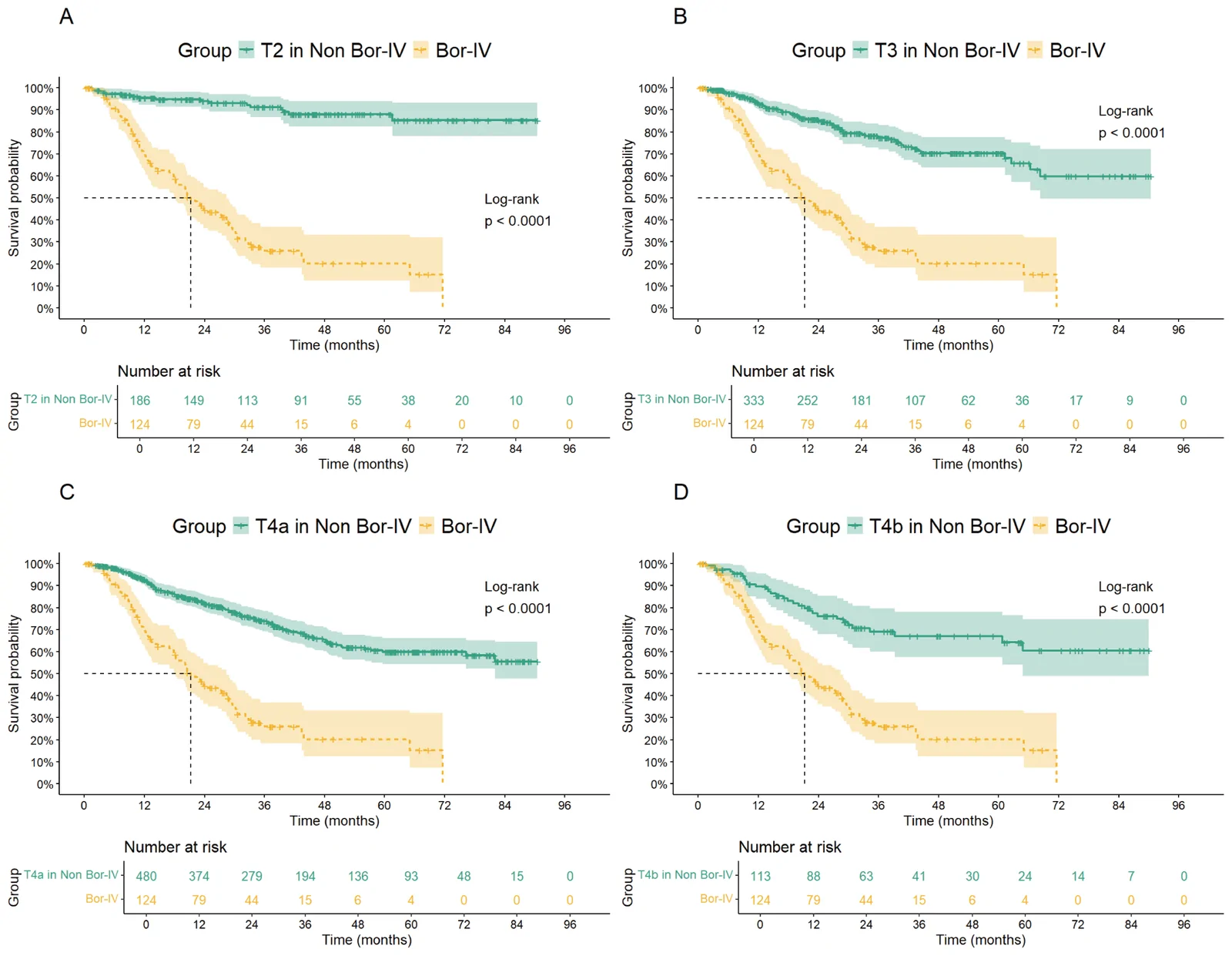
Stratified OS analysis between Bor-IV and Non Bor-IV groups according to T stage. **(A)** OS comparison between Bor-IV group and Non Bor-IV groups at T2 disease. **(B)** OS comparison between Bor-IV group and Non Bor-IV groups at T3 disease. **(C)** OS comparison between Bor-IV group and Non Bor-IV groups at T4a disease. **(D)** OS comparison between Bor-IV group and Non Bor-IV groups at T4b disease.

**Figure 5:**
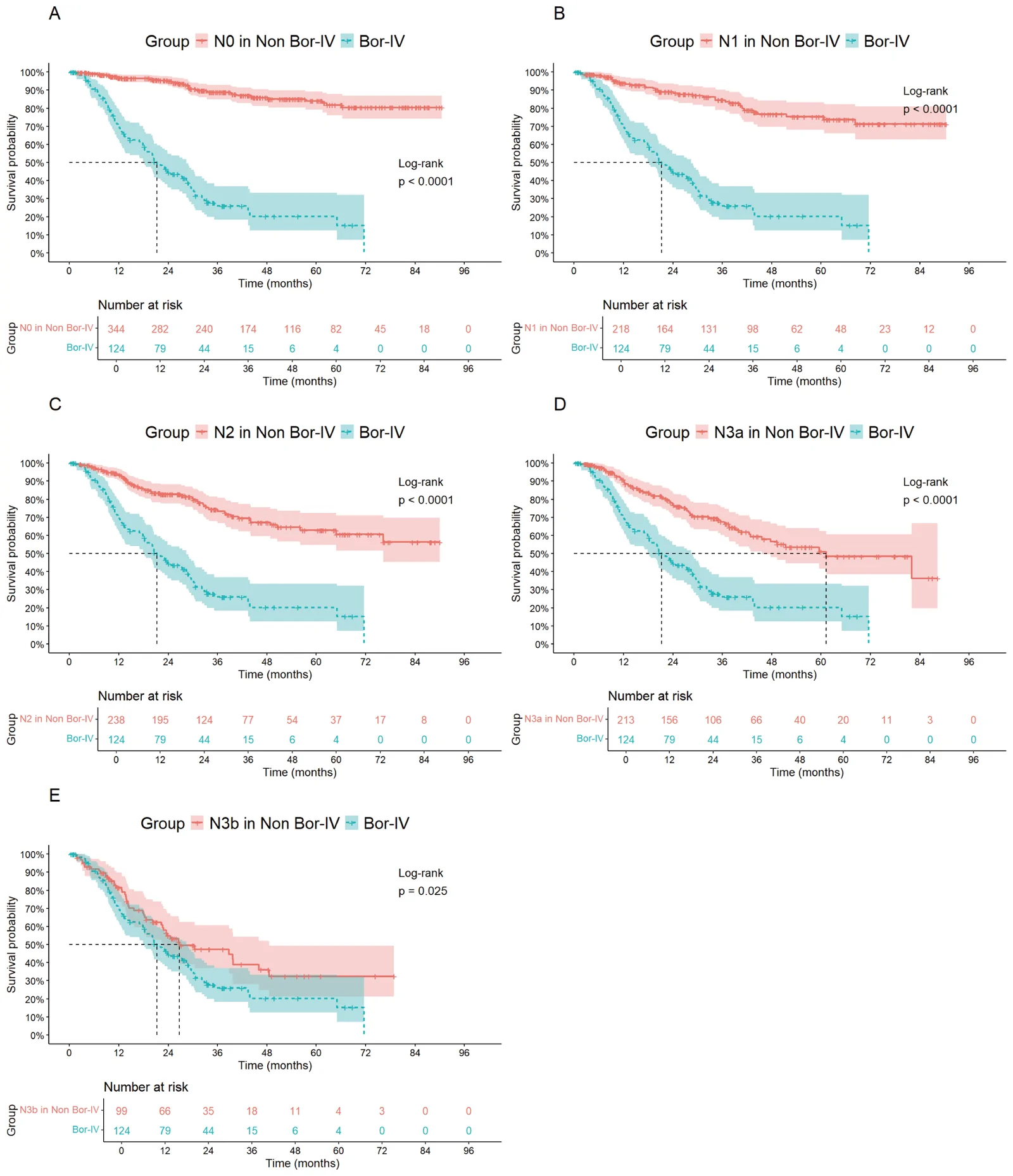
Stratified OS analysis between Bor-IV and Non Bor-IV groups according to N stage. **(A)** OS comparison between Bor-IV group and Non Bor-IV groups at N0 disease. **(B)** OS comparison between Bor-IV group and Non Bor-IV groups at N1 disease. **(C)** OS comparison between Bor-IV group and Non Bor-IV groups at N2 disease. **(D)** OS comparison between Bor-IV group and Non Bor-IV groups at N3a disease. **(E)** OS comparison between Bor-IV group and Non Bor-IV groups at N3b disease.

The median OS of Bor-IV patients was 21.3 months, with a 5-year OS rate of 20.24%, which was significantly worse than that of Non Bor-IV patients with T2 disease (5-year OS 87.99%), T3 disease (5-year OS 70.34%), T4a disease (5-year OS 59.98%), and T4b disease (5-year OS 67.09%) (all p < 0.001) (**Figure 4**). As for N stage, this survival disadvantage remained evident, as Bor-IV patients continued to demonstrate significantly poorer outcomes than Non Bor-IV patients with N0 disease (5-year OS 84.08%), N1 disease (5-year OS 75.34%), N2 disease (5-year OS 62.94%), N3a disease (median OS 61.3 months; 5-year OS 51.03%) and N3b disease (median OS 26.7 months; 5-year OS 32.43%) (all p < 0.05) (**Figure 5**).

These findings indicate that the prognostic burden associated with Bor-IV morphology exceeds that captured by conventional TNM staging alone, highlighting its exceptionally aggressive biological behavior.

### Impact of treatment modalities on prognosis in the Bor-IV group

**Figure 6:**
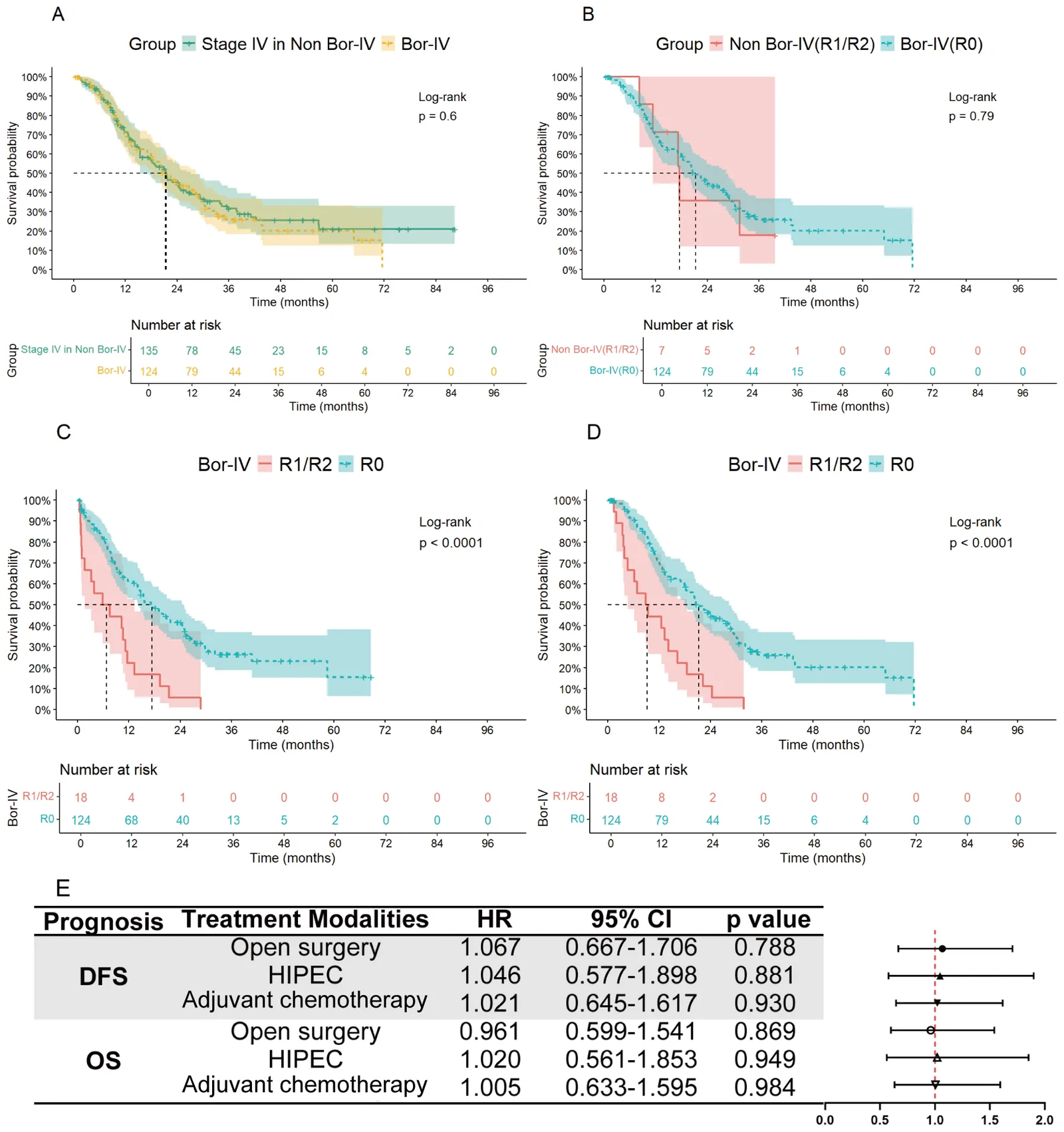
Survival outcomes of Bor-IV in comparison with stage IV disease and treatment-related prognostic factors. **(A)** OS comparison between Bor-IV and stage IV of Non Bor-IV. **(B)** No survival difference according to resection margin status between Bor-IV and Non Bor-IV. **(C, D)** DFS/OS comparison according to resection margin status in Bor-IV group. **(E)** Forest plots of treatment-related prognostic factors for DFS and OS.

Notably, survival outcomes of Bor-IV patients approached those observed in stage IV Non Bor-IV disease (median OS 21.5 months; 5-year OS 20.98%), suggesting that the biological aggressiveness of Bor-IV morphology confers a prognostic burden similar to that of distant metastatic disease (**Figure 6A**). And patients achieving negative margins in Bor-IV group (3-year OS 26.03%) exhibited similar survival compared with those with incomplete resection in Non Bor-IV group (median OS 17.5 months; 3-year OS 17.86%) (**Figure 6B**). With analysis of DFS and OS in Bor-IV group, patients achieving negative margins (median DFS 17.4 months, 1-year DFS 61.40% vs. median DFS 6.7 months, 1-year DFS 22.22%; median OS 21.3 months, 1-year OS 70.50% vs. median OS 9.2 months, 1-year OS 44.40%) exhibited better survival significantly (all p <0.001) (**Figures 6C, D**).

In Bor-IV patients with R0 resection, treatment-related prognostic analysis demonstrated limited survival benefit from therapeutic interventions. Forest plot analysis showed that open surgery, HIPEC, and postoperative chemotherapy were not significantly associated with either DFS or OS, with all confidence intervals crossing unity (**Figure 6E**).

## Discussion

Most patients with GC are diagnosed at an advanced stage, and despite the widespread adoption of multimodal treatment strategies centered on radical surgery, the long-term outcomes of AGC remain unsatisfactory. Within this heterogeneous disease spectrum, Bor-IV represents a distinct clinicopathological entity characterized by aggressive biological behavior and a poor prognosis. Although accumulating evidence has identified Bor-IV as an independent adverse prognostic factor in AGC, an effective and standardized comprehensive treatment strategy has yet to be established (5, 11, 20–22). Against this background, the present study systematically summarized the clinicopathological characteristics and survival patterns of Bor-IV and further explored the impact of different therapeutic modalities on patient outcomes, with the aim of providing clinically relevant evidence to guide individualized management.

In our present study, Bor-IV was found to be significantly associated with more advanced T stage, N stage, and TNM stage, suggesting that its poor prognosis may be partly attributable to higher tumor burden. However, multivariate Cox regression analysis demonstrated that Bor-IV remained an independent prognostic factor after adjusting for T stage, N stage, and other clinicopathological variables, indicating that the survival disadvantage cannot be fully explained by TNM stage alone and implying the presence of aggressive biological behaviors not adequately captured by the traditional staging system. Furthermore, multivariate analysis further identified advanced age and tumor depth (T stage) as the principal prognostic determinants in patients with Bor-IV. What is more, the survival of Bor-IV patients was significantly worse than that of Non Bor-IV patients with T4b disease, N3b disease, or stage IIIC, approximating the survival level of stage IV disease. However, this survival comparison does not imply biological equivalence, as Bor-IV is a macroscopic morphological classification, whereas stage IV represents established distant metastasis. Given that multiple subgroup comparisons were performed, some statistically significant results may be influenced by multiple testing. Therefore, these findings should be interpreted primarily as exploratory in nature and warrant further validation in larger, independent cohorts. Importantly, the primary conclusion-that Bor-IV is an independent prognostic factor-is derived from the multivariate Cox regression analysis, which is not subject to the same multiple-testing limitations as the subgroup comparisons. Notably, previous large-scale studies have suggested that conventional TNM staging may inadequately reflect the biological aggressiveness of this distinct subtype. For example, a cohort study involving 1,622 patients with AGC reported that OS in Bor-IV could be effectively stratified by pN classification and pathological TNM stage, but not by pT classification alone; consequently, the authors proposed classifying Bor-IV as pT4b disease to improve prognostic accuracy following radical surgery (23). Similarly, other studies have demonstrated that extensive lymph node metastasis—particularly pN3b disease—and large tumor size (>5 cm) are independent predictors of poor prognosis and peritoneal dissemination in Bor-IV (24). Importantly, even when compared with Non Bor-IV patients presenting with advanced pathological features such as T4b invasion and N3b nodal involvement, Bor-IV patients still exhibited inferior survival, which was comparable to that of stage IV disease in the Non Bor-IV group. Nonetheless, this observation should be interpreted with caution, as Bor-IV and stage IV disease are fundamentally different in their biological and clinical implications. These observations strongly suggest that Bor-IV possesses intrinsic malignant characteristics that are not adequately captured by current staging systems. Collectively, these findings support the concept that Bor-IV should be regarded as a distinct malignant entity within GC rather than merely a morphological subtype, underscoring the need for further large-scale, multicenter studies to refine its classification and optimize tailored therapeutic strategies.

In our present study, R0 resection emerged as the only intervention that significantly improved both DFS and OS in patients with Bor-IV. This finding is consistent with previous investigations emphasizing the critical importance of achieving complete tumor clearance (25–27). Accetta et al. reported that curative resection conferred a survival benefit in Bor-IV patients with stages IB-III disease, pN2 status, and localized tumors (28). In addition, large cohort studies from China and Korea consistently demonstrated superior outcomes in patients undergoing curative surgery compared with those receiving palliative resection or no surgery at all (10, 29). However, the infiltrative growth pattern characteristic of Borrmann type III and IV tumors poses substantial challenges in determining adequate surgical margins, often leading to microscopic residual disease. Consequently, extended gastrectomy has been advocated by some authors to improve the likelihood of achieving R0 resection, particularly in high-risk patients, who may also benefit from postoperative adjuvant chemotherapy (30). Conversely, other studies have suggested that in patients with large AGC, adjacent organ invasion, and extensive nodal involvement (N2-3), Bor-IV represents a biologically aggressive disease that may not be amenable to surgical cure, with limited survival benefit from resection alone (31). Despite these controversies, our data demonstrated that R0 resection significantly improved the 1-year OS to 70.5% in the Bor-IV group; however, this outcome remained similar to that observed in the R1/R2 subgroup of the Non Bor-IV group. These findings reinforce R0 resection as the cornerstone of treatment whenever technically feasible, while underscoring the urgent need for prospective trials to better define optimal surgical indications and perioperative strategies in this challenging patient population.

Given the advanced disease stage and high recurrence risk associated with Bor-IV, adjunctive treatment modalities have attracted increasing attention. In our cohort, neither adjuvant chemotherapy nor HIPEC was associated with a statistically significant survival benefit. These findings may reflect patient selection bias, heterogeneity in treatment regimens, and limited sample size, rather than the absence of therapeutic efficacy. Indeed, several previous studies have reported encouraging results with postoperative chemotherapy (32, 33). A Japanese study demonstrated improved survival outcomes with long-term postoperative S-1 chemotherapy in patients with Bor-IV (34), while a Chinese cohort study showed that subtotal gastrectomy combined with adjuvant chemotherapy not only improved prognosis but also reduced the incidence of postoperative peritoneal metastasis (13). With respect to HIPEC, as a locoregional cytoreductive strategy targeting microscopic peritoneal disease, its potential role in Bor-IV has been explored. A Korean study suggested that Bor-IV patients might represent suitable candidates for intraperitoneal chemotherapy among those with AGC (35), and a notable case report described a patient with Bor-IV and signet ring cell carcinoma who achieved a disease-free survival exceeding 61 months following cytoreductive surgery combined with HIPEC and both intraperitoneal and systemic chemotherapy (36). Although these findings are promising, the heterogeneity of existing data underscores the necessity for well-designed clinical trials to evaluate combined multimodal strategies specifically tailored to Bor-IV.

Some limitations of this study should be acknowledged. First, its retrospective, single-center design and relatively small sample size may introduce selection bias and limit the generalizability of the findings. Second, incomplete follow-up in a subset of patients may have affected survival analyses.

Third, patients receiving neoadjuvant therapy were excluded from our present study, as its inclusion would have confounded the assessment of surgical outcomes and baseline pathological staging. Neoadjuvant treatment can alter tumor pathology, making ypTNM stages non-equivalent to pTNM stages in treatment-naïve patients and compromising accurate prognostic interpretation. Moreover, the inherent heterogeneity of neoadjuvant regimens and responses would introduce substantial bias when combined with upfront surgery patients. Since one of our core objectives was to evaluate the impact of surgery on long-term survival in Bor-IV, including neoadjuvantly treated patients would make it difficult to isolate the survival benefit attributable to surgery alone. However, neoadjuvant therapy has become standard for AGC, and recent advances in chemo-immunotherapy have yielded substantial improvements in pathological response rates (pCR) (37–39). Emerging evidence suggests that novel neoadjuvant strategies may be especially valuable in Bor-IV (40). In China, preoperative epirubicin, etoposide, and oxaliplatin combined with oral S-1 (SEEOX) has been reported as a safe and effective regimen for Bor-IV, achieving a pCR of 33.3%, a median survival of 27.1 months, and a 2-year OS rate of 48.5% (41). Furthermore, a recent phase II trial demonstrated that perioperative PD-1 inhibitor combined with SOX chemotherapy significantly improved tumor regression in patients with Borrmann large type III and IV gastric cancer (42). These findings highlight the evolving therapeutic landscape and suggest that immunotherapy-based neoadjuvant approaches may enhance resectability and long-term outcomes in this historically refractory disease. Therefore, the conclusions of the present study are primarily applicable to GC patients undergoing direct surgery. And future large-scale studies incorporating patients receiving neoadjuvant and perioperative therapy are warranted to further refine the prognostic evaluation system for Bor-IV across different treatment modalities, and to provide more comprehensive evidence to support clinical decision-making. Fourth, our comparison of survival between Bor-IV and stage IV disease was intended to highlight the extremely poor prognosis of Bor-IV, not to imply biological equivalence. Bor-IV is a macroscopic morphological classification, whereas stage IV represents distant metastasis-these entities differ fundamentally, yet the comparison underscores the aggressive nature of Bor-IV and raises clinical awareness of its prognosis approaching that of stage IV disease.

## Conclusion

In this large single-center cohort, Bor-IV was consistently associated with markedly inferior survival compared with other macroscopic subtypes, even among patients with advanced TNM stages. The prognostic disadvantage of Bor-IV appeared to exceed that captured by conventional T and N classifications, and survival outcomes approached those observed in stage IV Non Bor-IV disease. These findings suggest that Bor-IV may represent a clinically aggressive phenotype that is inadequately reflected by current staging systems. Although achievement of R0 resection was associated with improved survival, outcomes remained poor, highlighting the urgent need for optimized perioperative strategies and novel multimodal treatment approaches. Prospective, multicenter studies incorporating contemporary chemotherapy and immunotherapy regimens are warranted to further validate these observations and to refine risk stratification and therapeutic decision-making for this high-risk subgroup.

## Data Availability

The original contributions presented in the study are included in the article/supplementary material. Further inquiries can be directed to the corresponding author.
