# Correction: Borrmann type IV gastric cancer exhibits distinct clinicopathological features and extremely poor prognosis

**DOI:** 10.3389/fonc.2026.1994613

**Published:** 2026-09-21

**Authors:** Qingyu Lin, Zikang Li, Wei Tang, Jianqiu Liu, Chanyan Huang, Ran Wei, Tianhao Zhang, Yan Qian, Yin Li, Jianhui Chen, Shirong Cai, Jinning Ye, Ertao Zhai

**Affiliations:** 1 Gastrointestinal Surgery Center, the First Affiliated Hospital of Sun Yat-sen University, Guangzhou, Guangdong, China; 2 Laboratory of Surgery, the First Affiliated Hospital of Sun Yat-sen University, Guangzhou, Guangdong, China; 3 Anesthesia Surgery Center, the First Affiliated Hospital of Sun Yat-sen University, Guangzhou, Guangdong, China

**Keywords:** advanced gastric cancer, Bormann classification, Bormann type IV gastric cancer, prognosis, R0 resection

An incorrect **Funding** statement was provided. The correct funder is “Guangdong Basic and Applied Basic Research Foundation”. The correct **Funding** statement reads:

“This work is supported by Guangdong Basic and Applied Basic Research Foundation (Grant No. 2023A1515111174 to Chanyan Huang)”.

The original version of this article has been updated.

